# Awareness of radiology staff in Saudi Arabia regarding patient privacy, ethical, and legal implications of sharing medical imaging on online platforms

**DOI:** 10.3389/fmed.2025.1677160

**Published:** 2025-11-12

**Authors:** Wejdan M. Arif

**Affiliations:** Radiological Sciences Department, College of Applied Medical Sciences, King Saud University, Riyadh, Saudi Arabia

**Keywords:** patient privacy, medical imaging, ethical implications, legal compliance, radiology awareness, Saudi Arabia

## Abstract

**Background:**

The digitization of radiology through online platforms has introduced significant challenges in maintaining patient privacy and complying with ethical and legal standards, particularly in Saudi Arabia’s rapidly evolving healthcare system.

**Aim:**

To assess the awareness levels of radiology professionals in Saudi Arabia regarding patient privacy, ethical responsibilities, and legal implications of sharing medical imaging on digital platforms.

**Methods:**

A cross-sectional survey was conducted among 293 radiology staff, including Radiologists and Radiologic Technologists, using a structured online questionnaire. Data were analyzed using descriptive statistics, *t*-tests, and ANOVA.

**Results:**

Participants demonstrated moderate awareness across all domains (mean scores ~3.1–3.2 on a 5-point scale). Radiologists reported significantly higher awareness than Technologists (*p* < 0.0001). Awareness also increased with years of experience and varied by region, with the Central region showing the highest scores.

**Conclusion:**

Despite moderate overall awareness, significant disparities highlight the need for targeted training and policy reinforcement to ensure consistent compliance with privacy, ethical, and legal standards in digital radiology.

## Introduction

The rapid digitization of radiology, driven by picture archiving and communication systems (PACS), telemedicine, and artificial intelligence (AI), has transformed diagnostic workflows but simultaneously introduced new challenges in safeguarding patient privacy and confidentiality (1–4). Within Saudi Arabia, healthcare digitization is expanding in alignment with Vision 2030 goals, where radiology departments increasingly rely on digital platforms for case discussions, second opinions, and multidisciplinary collaboration (5, 6). These technological advances, while beneficial, raise concerns about the ethical and legal management of sensitive medical images.

Patient privacy is defined as the individual’s right to control access to their health information, including medical images and associated metadata (7–9). This principle is central to maintaining patient trust and upholding professional integrity. Ethical responsibilities in radiology encompass obtaining informed consent, preventing unauthorized disclosures, and ensuring that educational or research uses of images respect patient autonomy (10). Legal frameworks further reinforce these obligations: in Saudi Arabia, the Personal Data Protection Law (PDPL), modeled on the EU’s General Data Protection Regulation (GDPR), sets strict requirements and penalties for unauthorized use or disclosure of patient data (9, 11). Medical imaging modalities such as MRI, CT, and X-rays carry inherent risks of re-identification, even when anonymized, due to identifiable features embedded in the images (12, 13).

Globally, the proliferation of social media and mobile platforms has blurred boundaries between clinical collaboration and public dissemination. Messaging applications like WhatsApp are widely used for rapid consultation, but recent studies demonstrate that even anonymized images can be re-identified with advanced software, raising doubts about the sufficiency of conventional anonymization practices (14, 15). In Saudi Arabia, where mobile health use surged following COVID-19, radiology staff face increased ethical and legal risks when sharing images without adequate safeguards (16, 17). The stakes are considerable: violations of the PDPL can result in fines of up to SAR 5 million (10, 18), while non-consensual sharing undermines ethical principles of autonomy and professional responsibility (10, 19).

Despite these challenges, evidence suggests that awareness among radiology professionals may be insufficient. For example, a 2023 Saudi study revealed that only 42% of radiology staff demonstrated proficiency in radiation safety, pointing to broader knowledge gaps in regulatory compliance and safe practice (16). Comparable deficiencies in privacy awareness are therefore plausible but remain underexplored.

Given this context, the present study aims to assess the awareness of radiology staff in Saudi Arabia regarding patient privacy, ethical responsibilities, and legal implications when sharing medical images on digital platforms. By identifying gaps in knowledge and highlighting areas of uncertainty, the findings will inform targeted training programs and institutional policies to enhance compliance with national regulations and international standards. This study therefore addresses a critical evidence gap in Saudi Arabia’s fast-evolving digital health landscape, with implications for patient trust, professional accountability, and regulatory enforcement (17, 20).

## Background

Recent advancements in medical imaging technology and data-sharing practices have intensified ethical, legal, and privacy challenges, particularly in radiology. Globally, the digitization of workflows has exposed vulnerabilities in conventional de-identification. A 2024 systematic review demonstrated that facial recognition software can re-identify up to 85% of individuals from brain MRI scans even after metadata removal, undermining traditional anonymization practices (21). While privacy-preserving approaches such as encryption and federated learning (FL) have shown promise—FL achieved 92% accuracy in pneumonia detection without exchanging raw data—adoption remains inconsistent, with only 35% of institutions worldwide enforcing encryption for cloud-based image sharing (22). Blockchain-based solutions have also been piloted for secure multi-institutional collaborations, but scalability and integration into hospital systems remain challenges (12, 23). These findings reveal a persistent gap between technical capabilities and real-world implementation.

Saudi Arabia’s healthcare system operates under a mixed administrative framework that includes public hospitals managed by the Ministry of Health, private facilities, military institutions, and academic medical centers. Radiology staff therefore function within diverse institutional environments, where policies and training resources may vary. Oversight mechanisms are provided by the Central Board for Accreditation of Healthcare Institutions (CBAHI), which enforces standards for patient safety and confidentiality, while the Saudi Commission for Health Specialties (SCFHS) governs professional licensing and continuing education. Such systems establish a nationwide baseline for quality but may not ensure uniform adoption of privacy-preserving practices (5, 6, 20).

The regulatory landscape in Saudi Arabia is multi-layered. The Saudi Constitution and the Basic Law of Governance enshrine general rights to privacy, while sectoral legislation provides more explicit protections. Most notably, the Personal Data Protection Law (PDPL), enacted in 2021, sets GDPR-like requirements for health data handling and imposes fines of up to SAR 5 million for unauthorized disclosures (11, 18). However, the PDPL does not operate in isolation. Accreditation requirements from CBAHI, codes of ethics from SCFHS, and sector-specific digital health guidelines collectively influence how institutions manage patient data. Studies reveal uneven compliance: Almuwail et al. (24) reported persistent privacy and security gaps in Saudi e-health systems, while Chikhaoui et al. (25) identified vulnerabilities in the use of cloud technologies for e-health. Similarly, Ukeje et al. (26) highlighted systemic risks in cloud adoption for government health systems, reinforcing concerns about Saudi Arabia’s reliance on digital infrastructure.

Comparative studies provide further insight into the maturity of Saudi Arabia’s regulatory environment. Sarabdeen and Ishak (27) noted differences between Saudi Arabia, Malaysia, and the EU in the scope and enforcement of privacy laws, emphasizing the relatively early stage of PDPL implementation. Al Khatib et al. (28) showed that even in UAE hospitals, aligning hospital management systems with GDPR remains a challenge, suggesting that Gulf countries face similar hurdles in operationalizing data protection frameworks. Within Saudi Arabia itself, barriers to effective data stewardship include inconsistent adoption of health data standards (29) and limited professional acceptance of advanced digital tools (30). These findings mirror broader international concerns that privacy protections often lag behind technological innovation.

At the same time, Saudi Arabia is pursuing ambitious digital health initiatives under Vision 2030. Artificial intelligence is increasingly positioned as a driver of sustainable healthcare, with studies identifying critical success factors for AI integration in the Saudi public health sector (31). However, without strong privacy safeguards, such technologies risk amplifying vulnerabilities. This echoes global findings: while homomorphic encryption and FL allow for secure analysis of encrypted data, their computational demands and reliance on high technical literacy have limited adoption in clinical practice (22, 23). Furthermore, most privacy-preserving technologies in radiology are still evaluated in controlled environments, with limited real-world validation (12). Patient engagement tools such as portals also remain underutilized; although international studies report growing patient interest in accessing imaging data, actual use remains low (32).

Overall, the Saudi health system presents a paradox. On one hand, reforms such as Vision 2030, accreditation systems, and the PDPL provide a robust framework for advancing digital healthcare. On the other, persistent evidence of institutional non-compliance, infrastructural weaknesses, and uneven training highlight a gap between policy and practice (33–37). These limitations underscore the importance of assessing how radiology staff—who are at the front line of image generation, sharing, and interpretation—understand and apply privacy, ethical, and legal requirements. By situating this study within both the global literature and Saudi Arabia’s unique health system and regulatory environment, it directly addresses a critical void in evidence on professional awareness in the Kingdom.

## Methods

### Study design

This study employed a cross-sectional design to assess the level of awareness among radiology staff in Saudi Arabia regarding patient privacy, as well as the ethical and legal implications of sharing medical imaging on online platforms. The study was conducted across various healthcare settings in the Kingdom of Saudi Arabia, encompassing a diverse range of institutions to ensure broad representation of radiology professionals. These settings included public hospitals under the Ministry of Health, private healthcare facilities, academic medical centers, and specialized diagnostic imaging clinics. The selected institutions were distributed across major regions of Saudi Arabia, including Riyadh, Jeddah, Dammam, and other key urban centers, to capture geographic diversity and variation in institutional practices.

### Recruitment and sampling

This study targeted radiology professionals working across various healthcare facilities in Saudi Arabia, including public hospitals, and private hospitals. Eligible participants included radiologists and radiologic technologists who are directly involved in the handling, processing, or dissemination of medical imaging data. Inclusion criteria required that participants be currently employed in a radiology-related role within the Kingdom of Saudi Arabia and possess at least 1 year of professional experience. Individuals not directly involved with medical imaging or those who declined to provide informed consent were excluded from the study.

A non-probability purposive sampling strategy (38) was employed to ensure the inclusion of participants with relevant professional backgrounds and exposure to digital medical imaging platforms. Recruitment was conducted through multiple channels, including professional networks, social media platforms (e.g., LinkedIn, Twitter), and official email distribution lists of radiology departments within healthcare institutions. Additionally, collaboration with professional bodies such as the Saudi Society of Radiology and the Saudi Commission for Health Specialties helped facilitate outreach to a wider pool of qualified professionals.

### Questionnaire design

The questionnaire designed for this study is structured into four main sections to comprehensively assess the awareness of radiology staff in Saudi Arabia regarding patient privacy, ethical, and legal implications of sharing medical imaging on online platforms. The first section gathers demographic information, including age, gender, job title, years of experience, institution type, region within Saudi Arabia, and prior training on patient privacy or data protection. The second section focuses on patient privacy awareness (9, 11, 16), presenting 10 statements related to knowledge and practices about safeguarding patient information, the importance of consent, institutional guidelines, and familiarity with privacy breach procedures; responses are rated on a 5-point Likert scale ranging from “Strongly Disagree” to “Strongly Agree.” The third section examines ethical implications (3, 8, 11), with 10 items assessing attitudes toward ethical responsibilities, professional guidelines, concerns about misuse, adequacy of ethical training, and awareness of ethical review processes. The final section addresses legal implications (17, 20, 37), probing familiarity with Saudi and international laws, understanding of legal consequences and penalties, institutional support and training, and knowledge of reporting mechanisms for legal violations. This comprehensive design ensures a thorough evaluation of radiology staff’s knowledge, attitudes, and practices regarding the responsible sharing of medical images in the digital age.

The questionnaire was translated from English to Arabic by a certified professional translator to ensure linguistic accuracy and clarity (39). To validate the quality and appropriateness of the translated version, two professors reviewed the Arabic draft. Their feedback focused primarily on grammatical refinements and linguistic precision, and their suggested revisions were incorporated to enhance the clarity and cultural relevance of the final version. Following the translation and expert validation, a pilot study was conducted with a group of six radiologists and two academic professors to assess the reliability and clarity of the questionnaire. The responses collected during this phase were analyzed using Cronbach’s alpha to evaluate internal consistency across the survey items. The resulting coefficient exceeded 0.7 (40), confirming that the questionnaire demonstrated a high level of reliability and was suitable for broader distribution within the target population.

### Data collection

Participants were invited to complete an anonymous, self-administered online questionnaire. The invitation included a cover letter explaining the study’s objectives, ethical considerations, and a consent form ensuring voluntary participation and data confidentiality. Data collection was conducted over a four-week period, during which reminder messages were periodically sent to maximize response rates.

### Data analysis

Data collected from the completed questionnaires was analyzed using the Statistical Package for the Social Sciences (SPSS) version 27 for. Descriptive statistics, including frequencies, percentages, means, and standard deviations, were used to summarize participants’ demographic characteristics and responses to each item related to privacy, ethical, and legal awareness. For the Likert-scale sections, mean scores were calculated for each respondent across the privacy, ethical, and legal domains to quantitatively represent their overall awareness levels. These mean scores served as the primary measure for comparing awareness among different subgroups. Inferential statistical tests were employed to explore associations between demographic variables (such as years of experience, professional role, and prior training) and mean awareness scores. Independent *t*-tests and one-way ANOVA compared mean scores across groups to assess the differences in awareness levels. A significance level of *p* < 0.05 was set for all statistical tests.

## Results

Table 1 presents the demographic distribution of the 293 radiology staff participants in Saudi Arabia. The age profile was relatively balanced, with the largest groups aged 31–40 (29.69%) and 51–60 (29.01%), followed by those aged 41–50 (27.99%) and 18–30 (13.31%). Gender representation was nearly equal, with males accounting for 50.51% and females 49.49%. Radiologic Technologists comprised the majority of the sample (63.48%), while Radiologists made up 36.52%. Participants’ work experience varied, with the largest groups having 1–3 years (27.99%) and 10 or more years (27.65%), while 7–9 years and 4–6 years of experience accounted for 25.94 and 18.43%, respectively. Regionally, the Northern (23.89%) and Central (22.18%) regions were most represented, followed by the Eastern (18.43%), Southern (18.09%), and Western (17.41%) regions. Institutional affiliations were also well distributed, with participants from public hospitals (27.65%), private hospitals (26.62%), military hospitals (23.21%), and university hospitals (22.53%). Notably, just under half (48.12%) reported having received formal training on patient privacy or data sharing, while the slight majority (51.88%) had not received formal training.

**Table 1:** Participants demographics.

Variables	Participants groups	*N*	Relative frequency
Age (in years)	18–30	39	13.31%
	31–40	87	29.69%
	41–50	82	27.99%
	51–60	85	29.01%
Gender	Male	148	50.51%
	Female	145	49.49%
Role	Radiologists	107	36.52%
	Radiologic Technologists	186	63.48%
Work experience	1 to 3	82	27.99%
	4 to 6	54	18.43%
	7 to 9	76	25.94%
	>=10	81	27.65%
Region	Central	65	22.18%
	Eastern	54	18.43%
	Northern	70	23.89%
	Southern	53	18.09%
	Western	51	17.41%
Type of hospital	Military hospital	68	23.21%
	Private hospital	78	26.62%
	Public hospital	81	27.65%
	University hospital	66	22.53%
Have you received formal training on patient privacy or data protection or data sharing principles?	Yes	141	48.12%
	No	152	51.88%

**Figure 1:**
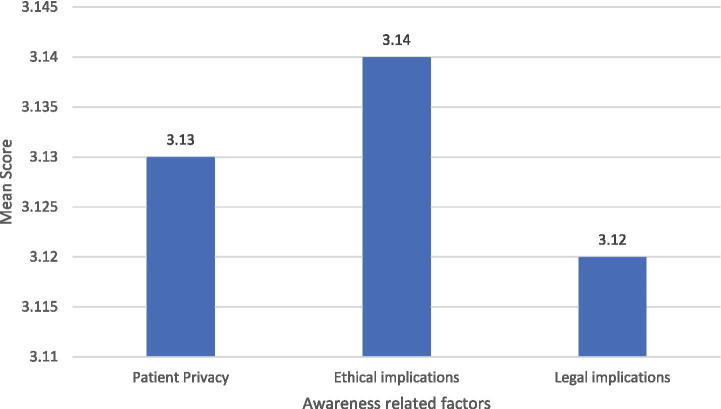
Mean awareness levels of the participants across the three domains (patient privacy, ethical implications, and legal implications).

The mean awareness scores across the three domains (see Figure 1) were closely aligned, with Patient Privacy (*M* = 3.13), Ethical Implications (*M* = 3.14), and Legal Implications (*M* = 3.12) all indicating moderate levels of awareness on a 5-point scale. These nearly identical averages suggest that radiology staff in Saudi Arabia demonstrate a balanced, though not high, understanding of privacy, ethical, and legal aspects of image sharing. The consistency across domains highlights that gaps in knowledge are systemic rather than confined to one particular area, underscoring the need for comprehensive training programs that simultaneously address all three dimensions (Figure 1).

Table 2 summarizes participants’ awareness levels regarding patient privacy when sharing medical images online. Overall, the mean scores across the 10 items ranged narrowly from 3.08 to 3.19 on a 5-point Likert scale, indicating a moderate level of awareness among radiology staff. The highest reported awareness was in regularly updating knowledge of privacy regulations (*M* = 3.19, SD = 1.00) and ensuring identifiers are removed before sharing images (*M* = 3.15, SD = 1.00). Participants also showed relatively consistent awareness regarding the need to obtain patient consent (*M* = 3.12, SD = 1.03), the potential risks of sharing anonymized images (*M* = 3.10, SD = 0.98), and the availability of institutional guidelines (*M* = 3.10, SD = 0.99). However, awareness remained modest across all items, reflecting neither strong compliance nor critical gaps but rather a medium level of understanding and engagement with patient privacy practices in digital radiology contexts. The standard deviations, all close to 1.0, suggest a fair amount of variability in responses, indicating differences in individual awareness levels within the participant group.

**Table 2:** Participants awareness levels of patients privacy.

Items	Mean score (*M*)	Standard deviation (SD)
I am aware that sharing medical images online can compromise patient privacy.	3.13	1.05
I always ensure that patient identifiers are removed before sharing medical images.	3.15	1.00
I understand the importance of obtaining patient consent before sharing their medical images online.	3.12	1.03
My institution provides clear guidelines on protecting patient privacy when sharing images.	3.10	0.99
I feel confident in my ability to safeguard patient privacy in digital environments.	3.14	0.95
I believe that sharing anonymized images online still carries privacy risks.	3.10	0.98
I am familiar with the procedures for reporting privacy breaches in my workplace.	3.15	0.98
I regularly update my knowledge regarding patient privacy regulations.	3.19	1.00
I believe that patient privacy should take priority over educational or research benefits when sharing images.	3.08	0.96
I am aware of recent incidents where patient privacy was compromised due to online image sharing.	3.11	1.02

Table 3 presents participants’ awareness levels regarding the ethical implications of sharing medical images online. The mean scores for all 10 items ranged from 3.10 to 3.18 on a 5-point scale, indicating a moderate overall awareness of ethical responsibilities. The highest mean was observed for concern about the potential misuse of shared images (*M* = 3.18, SD = 1.01) and awareness of ethical review processes such as IRB approvals (*M* = 3.17, SD = 1.02), reflecting participants’ recognition of formal oversight mechanisms. Similarly, participants expressed a strong sense of professional responsibility to maintain patient confidentiality (*M* = 3.16, SD = 0.97) and acknowledged the importance of ethical training (*M* = 3.16, SD = 1.02). Awareness of ethical guidelines from professional bodies and the justifiability of image sharing for educational purposes if privacy is protected both had mean scores of 3.11, suggesting moderate familiarity with these considerations. While participants generally acknowledged the importance of ethical practices, the data also hint at inconsistencies, with lower scores for perceptions of overlooked ethics in favor of convenience (*M* = 3.10, SD = 1.00). Overall, the responses reflect a medium level of ethical awareness with variability in depth of understanding, as suggested by standard deviations close to 1.00.

**Table 3:** Participants awareness levels of ethical implications.

Items	Mean score (*M*)	Standard deviation (SD)
I consider the ethical implications before sharing any medical image online.	3.13	1.02
I believe it is unethical to share medical images without explicit patient consent.	3.13	0.99
I feel a professional responsibility to always protect patient confidentiality.	3.16	0.97
I am aware of the ethical guidelines set by professional radiology organizations regarding image sharing.	3.11	1.03
I believe that sharing images online for educational purposes can be justified if privacy is protected.	3.11	0.98
I am concerned about the potential misuse of medical images shared online.	3.18	1.01
I have discussed ethical dilemmas related to image sharing with colleagues or supervisors.	3.14	0.96
I feel that current ethical training on image sharing is sufficient.	3.16	1.02
I believe that ethical considerations are sometimes overlooked in favor of convenience or speed.	3.10	1.00
I am aware of ethical review processes (e.g., IRB) required before sharing images for research.	3.17	1.02

Table 4 outlines participants’ awareness levels concerning the legal implications of sharing medical images online. Mean scores for the 10 items ranged narrowly from 3.10 to 3.15, indicating a generally moderate level of legal awareness among radiology staff. The highest awareness was reported for the belief that legal requirements are clearly communicated to staff (*M* = 3.15, SD = 1.03), followed closely by familiarity with institutional training on legal aspects (*M* = 3.14, SD = 0.98), awareness of international regulations like HIPAA (*M* = 3.14, SD = 1.04), and knowledge of recent legal cases involving image sharing (*M* = 3.14, SD = 1.01). Participants also demonstrated moderate understanding of national laws (*M* = 3.12, SD = 1.03) and the legal consequences of unauthorized sharing (*M* = 3.12, SD = 1.00). Confidence in institutional compliance (*M* = 3.10, SD = 1.03) and awareness of reporting mechanisms (*M* = 3.11, SD = 1.05) showed slightly lower but consistent levels of awareness. Standard deviations close to or slightly above 1.00 suggest moderate variability in legal knowledge among respondents. Overall, the findings indicate that while legal awareness is present, it remains at a medium level, with room for improvement through more targeted legal education and policy communication.

**Table 4:** Participants awareness levels of legal implications.

Items	Mean score (*M*)	Standard deviation (SD)
I am familiar with Saudi laws regarding the sharing of medical images online.	3.12	1.03
I understand the legal consequences of unauthorized sharing of patient images.	3.12	1.00
My institution provides training on the legal aspects of patient data sharing.	3.14	0.98
I am aware of the penalties for violating patient privacy laws in Saudi Arabia.	3.10	1.04
I know how to seek legal advice if I am unsure about sharing medical images.	3.11	0.99
I am familiar with international regulations (e.g., HIPAA) that influence local practices.	3.14	1.04
I feel confident that my workplace complies with all relevant legal requirements for image sharing.	3.10	1.03
I am aware of recent legal cases involving improper sharing of medical images.	3.14	1.01
I believe that legal requirements are clearly communicated to all radiology staff.	3.15	1.03
I am aware of the process to report legal violations related to patient data sharing.	3.11	1.05

Table 5 reveals statistically significant differences in awareness levels between Radiologists and Radiologic Technologists across all three domains—patient privacy, ethical implications, and legal implications (*p* < 0.0001). Radiologists consistently reported higher mean awareness scores (Patient Privacy: *M* = 4.03; Ethical: *M* = 4.04; Legal: *M* = 4.06) compared to Radiologic Technologists (Patient Privacy: *M* = 2.61; Ethical: *M* = 2.62; Legal: *M* = 2.58). These differences, coupled with relatively low variances, indicate a strong and consistent trend of greater awareness among Radiologists, potentially reflecting their advanced training, higher responsibility levels, or broader exposure to regulatory standards in clinical practice.

**Table 5:** Differences in participants perceptions (by role, work experience, type of hospital, and region) in relation the awareness of patient privacy, ethical, and legal implications using ANOVA.

Variables	Awareness factors	Participants groups	*N*	Mean (*M*)	Variance	*p*-value
Role	Patient privacy	Radiologists	107	4.03	0.24	<0.0001*
		Radiologic Technologists	186	2.61	0.23	
	Ethical implications	Radiologists	107	4.04	0.22	<0.0001*
		Radiologic Technologists	186	2.62	0.24	
	Legal implications	Radiologists	107	4.06	0.24	<0.0001*
		Radiologic Technologists	186	2.58	0.25	
Work experience	Patient privacy	1 to 3	82	2.61	0.53	<0.0001*
		4 to 6	54	3.16	0.59	
		7 to 9	76	3.16	0.61	
		>=10	81	3.60	0.56	
	Ethical implications	1 to 3	82	2.63	0.57	<0.0001*
		4 to 6	54	3.16	0.61	
		7 to 9	76	3.15	0.59	
		>=10	81	3.63	0.54	
	Legal implications	1 to 3	82	2.58	0.61	<0.0001*
		4 to 6	54	3.19	0.65	
		7 to 9	76	3.17	0.57	
		>=10	81	3.59	0.62	
Type of hospital	Patient privacy	Military hospital	68	3.08	0.69	0.6368
		Private hospital	78	3.22	0.83	
		Public hospital	81	3.14	0.73	
		University hospital	66	3.05	0.55	
	Ethical implications	Military hospital	68	3.04	0.74	0.4781
		Private hospital	78	3.25	0.80	
		Public hospital	81	3.15	0.69	
		University hospital	66	3.10	0.58	
	Legal implications	Military hospital	68	3.04	0.75	0.3938
		Private hospital	78	3.24	0.88	
		Public hospital	81	3.16	0.71	
		University hospital	66	3.02	0.65	
Region	Patient privacy	Central	65	3.52	0.59	<0.0001*
		Eastern	54	3.36	0.61	
		Northern	70	2.96	0.66	
		Southern	53	2.69	0.62	
		Western	51	3.06	0.66	
	Ethical implications	Central	65	3.53	0.56	<0.0001*
		Eastern	54	3.37	0.57	
		Northern	70	2.99	0.73	
		Southern	53	2.72	0.61	
		Western	51	3.02	0.67	
	Legal implications	Central	65	3.56	0.64	<0.0001*
		Eastern	54	3.35	0.64	
		Northern	70	2.91	0.68	
		Southern	53	2.78	0.67	
		Western	51	2.97	0.73	

*Statistically significant difference.

Significant differences were also observed across work experience groups for all three awareness factors (*p* < 0.0001). Participants with greater experience (≥10 years) demonstrated higher awareness in patient privacy (*M* = 3.60), ethical implications (*M* = 3.63), and legal implications (*M* = 3.59), compared to those with 1–3 years of experience (Patient Privacy: *M* = 2.61; Ethical: *M* = 2.63; Legal: *M* = 2.58). This gradient suggests that awareness grows with professional exposure and tenure, possibly due to accumulated training, institutional familiarity, and encounters with privacy-related challenges over time.

No statistically significant differences were found in awareness levels based on the type of hospital (Patient Privacy: *p* = 0.6368; Ethical Implications: *p* = 0.4781; Legal Implications: *p* = 0.3938). While mean scores varied slightly—for instance, private hospital staff showed marginally higher awareness in legal aspects (*M* = 3.24) than those in military (*M* = 3.04) or university hospitals (*M* = 3.02)—these variations were not statistically meaningful. This suggests that awareness training and institutional emphasis on privacy, ethics, and legality may be comparably implemented across different hospital types in Saudi Arabia.

Significant regional differences were found in participants’ awareness across all three domains (*p* < 0.0001). The Central region consistently showed the highest mean scores (Patient Privacy: *M* = 3.52; Ethical: *M* = 3.53; Legal: *M* = 3.56), followed by the Eastern region. In contrast, the Southern and Northern regions displayed the lowest awareness levels, particularly in legal implications (Southern: *M* = 2.78; Northern: *M* = 2.91). These findings suggest geographic disparities in awareness, possibly reflecting unequal access to training resources, institutional policies, or regional prioritization of data protection standards.

## Discussion

The results of this study indicate a moderate level of awareness among radiology staff in Saudi Arabia regarding patient privacy, ethical responsibilities, and legal implications when sharing medical images online. Across all three domains, mean scores ranged narrowly between 3.08 and 3.19, suggesting that while participants possess foundational knowledge, there remains a substantial opportunity for improvement. This finding mirrors previous regional studies that reported similar deficiencies in radiology-related safety practices, with only 42% of Saudi radiology staff demonstrating proficiency in radiation safety protocols (16), implying parallel gaps in data privacy and ethical compliance. While most participants acknowledged the importance of consent and confidentiality, ethical dilemmas persist in educational and research contexts, where images are sometimes shared for learning without explicit patient authorization. For example, anonymized radiographs may be posted in academic forums without clear safeguards, raising concerns about residual identifiability. Incorporating qualitative research or case-based training could provide richer insight into how professionals navigate these dilemmas in practice.

Findings also revealed that while most participants understood the basic principles of patient privacy and the importance of informed consent, significant gaps remain in the practical application of these concepts and in familiarity with specific legal frameworks such as the Saudi PDPL. Radiologists consistently demonstrated significantly higher awareness than Radiologic Technologists across patient privacy (*M* = 4.03 vs. 2.61), ethical (*M* = 4.04 vs. 2.62), and legal domains (*M* = 4.06 vs. 2.58) (Table 5). This discrepancy is likely due to differences in educational background, clinical responsibilities, and exposure to regulatory standards. A key finding is that radiology staff who had received formal training in data protection demonstrated significantly higher awareness scores across all domains. This aligns with earlier regional studies, which also identified education and training as critical determinants of compliance with privacy and data protection protocols (16, 24). However, the overall proportion of staff with such training was suboptimal, suggesting that current institutional efforts in Saudi Arabia may not be sufficient to ensure widespread competency in this area. This study focused on awareness and knowledge; however, integrating data on actual compliance behaviors and incident reporting related to privacy breaches would strengthen understanding of how awareness translates into practice. Future studies should consider linking self-reported awareness with objective measures of compliance and breach incidents to provide a comprehensive evaluation.

When compared to international literature, the results are consistent with global trends highlighting the inadequacy of traditional anonymization methods in the face of advancing re-identification technologies. As referenced in the background, up to 85% of facial reconstructions from MRI scans can be re-identified, even after attempts at de-identification (9, 21). In this study, many participants underestimated these risks, reflecting a gap between theoretical knowledge and awareness of emerging threats. This is particularly concerning given the increasing use of digital platforms for clinical collaboration, education, and research, as well as the rapid digital transformation under Saudi Arabia’s Vision 2030 (6, 17).

Ethical awareness was generally high, with most respondents recognizing the necessity of obtaining patient consent and the importance of confidentiality. Nevertheless, a substantial minority expressed uncertainty regarding the ethical boundaries of image sharing, particularly for educational or research purposes—a finding consistent with (36) where 68% of radiologists admitted to sharing images via messaging platforms without explicit consent. This highlights the persistent tension between clinical efficiency and ethical rigor, as well as the need for clearer institutional policies and professional guidelines. Legal awareness was the lowest among the three domains, with many participants unfamiliar with the specific penalties and reporting mechanisms mandated by Saudi PDPL and related regulations. Despite the enactment of the Saudi PDPL, participants’ low legal awareness suggests challenges in implementation, including limited dissemination of regulations, insufficient institutional legal guidance, and the novelty of PDPL compliance frameworks. Strengthening legal education through workshops, inclusion of PDPL modules in professional training, and active monitoring by regulatory authorities could help bridge this gap and enhance proactive compliance. This finding is in line with previous reports that legal compliance is often reactive, with staff more knowledgeable about breach reporting than about proactive legal safeguards (18, 36). Furthermore, awareness of cross-border data sharing regulations and the use of patient portals for transparency and education was limited, echoing international concerns about the underutilization of these tools (32, 37).

The results also indicate that professional experience plays a crucial role in shaping awareness. Staff with 10 or more years of experience reported significantly higher scores across all domains compared to those with one to 3 years (*p* < 0.0001). This trend aligns with findings from global surveys, which demonstrate that long-term clinical exposure enhances familiarity with data protection procedures and ethical reasoning (33, 35).

Geographic location emerged as another key determinant of awareness. Participants from the Central region had the highest scores in all three domains, while those from the Southern and Northern regions had the lowest. These results suggest regional disparities in access to training or institutional support. Given Saudi Arabia’s ongoing digital health transformation under Vision 2030, such variations raise concerns about equitable implementation of privacy protocols nationwide (6). Although privacy-preserving technologies show promise, practical adoption faces barriers including high implementation costs, challenges integrating new tools into existing workflows, and limited staff familiarity and training. Addressing these obstacles is crucial for effective deployment within radiology departments.

The finding that fewer than half of participants had received formal training highlights systemic barriers, such as limited institutional resources, uneven access to continuing education, competing clinical priorities, and gaps in institutional policies. To address these gaps, scalable solutions—such as mandatory online training modules, integration of privacy curricula in professional licensing requirements, and collaboration with national bodies like the Saudi Commission for Health Specialties—are recommended to ensure equitable access to training across regions and hospital types. Despite the presence of national regulations such as the PDPL, which mirrors the GDPR and carries fines up to SAR 5 million for breaches (18), the awareness of legal consequences remained modest, suggesting poor regulatory dissemination or ineffective training. In conclusion, although awareness among Saudi radiology staff is not alarmingly low, the evident differences by role, experience, and region highlight the need for targeted interventions to ensure consistent and comprehensive understanding of privacy, ethical, and legal obligations across the radiology workforce. Efforts to increase awareness of the PDPL and related laws should include comprehensive dissemination strategies, such as institutional workshops and integration into professional licensing. Comparative insights from other countries with mature data protection laws may guide these efforts. Additionally, investigating patient attitudes toward image sharing and privacy could align healthcare practices with patient expectations, fostering trust and transparency. Given the increasing use of global digital platforms, cross-border data sharing poses significant privacy and legal risks, including compliance with multiple jurisdictions. Institutional safeguards such as data localization policies, secure transfer protocols, and strict access controls are essential to mitigate these risks.

Based on these findings, it is recommended that healthcare institutions in Saudi Arabia implement mandatory, recurring training programs focused on patient privacy, ethical image sharing, and legal compliance, with content tailored to local regulations and real-world scenarios, and also tailored to both Radiologists and Technologists. Ethical challenges in sharing images for education and research include balancing educational benefits with patient privacy and consent. Developing clear institutional policies should be developed to address both traditional and emerging risks, including social media use and cross-border data sharing. Investment in privacy-preserving technologies must be accompanied by practical training and workflow integration. Focusing on regional capacity building, resources should be directed to underperforming regions (e.g., Southern and Northern Saudi Arabia) to close geographic awareness gaps. Furthermore, clear institutional protocols and reporting mechanisms must be communicated, with visible consequences for non-compliance. In addition, privacy-preserving technologies (e.g., FL, blockchain) should be incorporated into routine workflows alongside practical training for staff. Finally, further research should explore patient perspectives, the effectiveness of educational interventions, and the impact of new technologies on privacy practices in the Saudi context. By addressing these gaps, Saudi Arabia can strengthen its digital health infrastructure while maintaining patient trust and upholding the highest standards of ethical and legal practice.

### Implications

Theoretically, this study advances the understanding of how awareness of patient privacy, ethical, and legal considerations is distributed among radiology staff in the context of Saudi Arabia’s rapidly digitizing healthcare sector, highlighting the persistent gap between general knowledge and practical application, especially regarding nuanced legal frameworks like the PDPL and the risks associated with advanced re-identification technologies. Practically, the results underscore the urgent need for context-specific, mandatory, and recurring training programs tailored to local regulations and real-world scenarios, as staff with prior data protection training demonstrated significantly higher awareness levels, and many participants showed uncertainty about ethical boundaries and legal compliance in daily practice; this calls for clear institutional guidelines, robust reporting mechanisms, and integration of privacy-preserving technologies into clinical workflows. Emerging privacy-preserving technologies, such as federated learning and blockchain, offer promising solutions but face barriers to real-world adoption. These include high computational demands, lack of interoperability with existing hospital systems, and limited staff familiarity with advanced AI tools. Pilot programs and phased integration—combined with staff training—are needed before these innovations can be effectively scaled in Saudi radiology practice. Furthermore, the findings point to the necessity for healthcare organizations to invest in both technical solutions and continuous professional development, as well as to expand patient education and engagement through secure portals, thereby not only mitigating the risk of data breaches but also strengthening patient trust and upholding professional and regulatory standards in line with Saudi Arabia’s Vision 2030.

### Limitations

This study has several limitations that should be considered when interpreting the findings. First, the use of a cross-sectional design and self-administered questionnaire may introduce response bias, as participants might overestimate their awareness or provide socially desirable answers rather than reflecting actual practices. This study employed non-probability purposive sampling, which may limit generalizability since participants who are more aware of privacy and legal issues might have been more inclined to respond. Future studies should adopt randomized, nationally representative sampling to enhance the generalizability of findings and reduce the likelihood of selection bias, thereby capturing a more accurate reflection of the Saudi radiology workforce. While this study presents valuable quantitative insights into awareness levels, future research could benefit from incorporating qualitative methods such as interviews or focus groups. Such approaches would enable deeper exploration of how radiology professionals navigate ethical dilemmas and privacy challenges in practice, thus enriching the understanding of awareness-behavior gaps and informing tailored interventions.

## Conclusion

This study highlights a moderate overall level of awareness among radiology professionals in Saudi Arabia regarding patient privacy, ethical responsibilities, and legal obligations associated with sharing medical imaging on online platforms. Significant differences were observed based on professional role, years of experience, and geographic region, with Radiologists, more experienced staff, and those in the Central region demonstrating higher awareness levels. These findings underscore the need for targeted, role-specific training and policy reinforcement to ensure consistent understanding and application of privacy regulations across all levels of radiology practice. As Saudi Arabia continues to advance its digital health infrastructure under Vision 2030, enhancing institutional support, promoting equitable access to training, and integrating privacy-preserving technologies are essential for safeguarding patient data and upholding ethical and legal standards in digital radiology environments.

## Data Availability

The raw data supporting the conclusions of this article will be made available by the authors, without undue reservation.

## References

[1] Arpaci I Barzegari S Askarian F. Adoption of picture archiving and communication system (PACS) by healthcare professionals In Proceedings of International Conference on Emerging Technologies and Intelligent Systems. ICETIS 2021. Lecture notes in networks and systems. (Eds.) Al-Emran, M., Al-Sharafi, M.A., Al-Kabi, M.N., and Shaalan, K. Cham: Springer (2021). 807–13.

[2] Tadayon H Nafari B Khadem G Darrudi R Jabali MS. Evaluation of picture archiving and communication system (PACS): radiologists’ perspective. Inf Med Unlocked. (2023) 39:101266. doi: 10.1016/j.imu.2023.101266

[3] Van Leeuwen KG Schalekamp S Rutten MJCM van Ginneken B de Rooij M. Artificial intelligence in radiology: 100 commercially available products and their scientific evidence. Eur Radiol. (2021) 31:3797–804. doi: 10.1007/s00330-021-07892-z, PMID: 33856519 PMC8128724

[4] Vasilev YA Kozhikhina DD Vladzymyrskyy AV Shumskaya YF Mukhortova AN Blokhin IA . Results of the work of the reference center for diagnostic radiology using telemedicine technology. Health Care Russian Feder. (2024) 68:102–8. doi: 10.47470/0044-197X-2024-68-2-102-108

[5] Abdullah AM Al-Zughaibi N Al-Harbi FS Al Dhaheri MAD. Digital health integration in radiological screening practices: opportunities and challenges for technicians in Saudi vision 2030. J Int Crisis Risk Commun Res. (2024) 7:2499

[6] Aldawsari NSM Alharbi NMS. Saudi vision 2030 and physician’s perception of public-private partnerships in healthcare. Open Access Public Health Administrat Rev. (2023) 2:18–31. doi: 10.59644/oaphhar.2(1).41

[7] Herington J McCradden MD Creel K Boellaard R Jones EC Jha AK . Ethical considerations for artificial intelligence in medical imaging: deployment and governance. J Nucl Med. (2023) 64:1509–15. doi: 10.2967/jnumed.123.266110, PMID: 37620051 PMC12782051

[8] Kaissis G Ziller A Passerat-Palmbach J Ryffel T Usynin D Trask A . End-to-end privacy preserving deep learning on multi-institutional medical imaging. Nat Mach Intell. (2021) 3:473–84. doi: 10.1038/s42256-021-00337-8

[9] Royal College of Radiologists. (2019). Guidance on maintaining patient confidentiality when using radiology department information systems (2nd ed.). Royal College of Radiologists. Available online at: https://www.rcr.ac.uk/media/slmbkf0d/rcr-publications_guidance-on-maintaining-patient-confidentiality-when-using-radiology-department-information-systems-second-edition_november-2019.pdf (accessed Sep 15, 2025)

[10] Segal JP Hansen R. Medical images, social media and consent. Nat Rev Gastroenterol Hepatol. (2021) 18:517–8. doi: 10.1038/s41575-021-00453-1, PMID: 33893456 PMC8063782

[11] Klenske N. (2021). Protecting patient privacy in the era of artificial intelligence. RSNA News. Available online at: https://www.rsna.org/news/2021/february/protecting-patient-privacy (accessed Feb 12, 2025)

[12] Beryl JR Sood A Pattnaik T Malhotra R Nayyar V Narayan B . Medical imaging privacy: a systematic scoping review of key parameters in dataset construction and data protection. J Med Imaging Radiat Sci. (2025) 56:101914. doi: 10.1016/j.jmir.2025.101914 40288182

[13] Clunie D. A. Flanders A. Taylor A. Erickson B. Bialecki B. Brundage D. . Report of the medical image de-identification (MIDI) task group: best practices and recommendations. arXiv. (2025). Available online at: https://arxiv.org/abs/2303.10473v3 (Accessed Mar 16, 2025)

[14] Paul J. (2024). Cloud-based healthcare platforms: bridging the gap in rural and urban health services. ResearchGate Available online at: https://www.researchgate.net/profile/Lorenzaj-Harris/publication/387425231 [accessed Mar 16, 2025]

[15] Singh K. Artificial intelligence & cloud in healthcare: analyzing challenges and solutions within regulatory boundaries. Int J Comput Sci Engineer. (2023) 10:1–9. doi: 10.14445/23488387/ijcse-v10i9p101

[16] Al-Shahrani LAM Al-Amri MD Hufayyan RSA Qahtani AAA Al-Shahrani TK. Assessing the knowledge of radiology staff on the impact of radiation exposure and the importance of shielding for patient protection in Saudi Arabia. Int J Adv Human Res. (2024) 4:1–15. doi: 10.21608/ijahr.2024.327660.1046

[17] Mahajan R. Posting clinical data and images on social media: ethical and legal considerations. Adesh Univ J Med Sci Res. (2023) 5:1–4. doi: 10.25259/AUJMSR_29_2023

[18] Usercentrics GmbH, U. (2023). Saudi Arabia personal data protection law (PDPL): An overview. Usercentrics. Available online at: https://usercentrics.com/knowledge-hub/saudi-arabia-personal-data-protection-law-pdpl (accessed Mar 16, 2025)

[19] Pugh J. Autonomy, rationality, and contemporary bioethics [internet]. Oxford (UK): Oxford University Press (2020). Available at: https://www.ncbi.nlm.nih.gov/books/NBK556857/32396289

[20] Desert Valley Radiology. (2025). Ethical considerations in medical imaging. Available online at: https://www.dvrphx.com/ethical-considerations-in-medical-imaging.html (accessed March 16, 2025)

[21] Radiology Today. (2019). Mayo Clinic studies patient privacy in MRI research. Radiology today news. Available at: https://www.radiologytoday.net/news/103119_news.shtml (accessed March 16, 2025)

[22] Zhu Y. Yin X. Liew A. W. C. Tian H. (2024). Privacy-preserving in medical image analysis: a review of methods and applications. arXiv. Available at: http://arxiv.org/abs/2412.03924 (accessed March 16, 2025)

[23] Boulila W. Ammar A. Benjdra B. Koubaa A. Securing the Classification of COVID-19 in Chest X-ray Images: A Privacy-Preserving Deep Learning Approach. 2022 2nd International Conference of Smart Systems and Emerging Technologies (SMARTTECH), Riyadh, Saudi Arabia, [Internet]. (2022) 220–5. doi: 10.1109/smarttech54121.2022.00055

[24] Almuwail KI Albarrak AS Nasir M Bhutta M Wahsheh HA. Examining the factors for non-compliance of Saudi health organizations for e-health security and privacy. J Theor Appl Inf Technol. (2023) 101:435–47.

[25] Chikhaoui E Sarabdeen J Parveen R. Privacy and security issues in the use of clouds in e-health in the Kingdom of Saudi Arabia. Commun IBIMA. (2017) 2017:1–18. doi: 10.5171/2017.369309

[26] Ukeje N Gutierrez J Petrova K. Information security and privacy challenges of cloud computing for government adoption: a systematic review. Int J Inf Secur. (2024) 23:1459–75. doi: 10.1007/s10207-023-00797-6

[27] Sarabdeen J Ishak MM. A comparative analysis: health data protection laws in Malaysia, Saudi Arabia and EU general data protection regulation (GDPR). Int J Law Manag. (2025) 67:99–119. doi: 10.1108/IJLMA-01-2024-0025

[28] Al Khatib I Ahmed N Ndiaye M. GDPR compliance of hospital management systems in the UAE. J Data Sci Intell Syst. (2024):1–14. doi: 10.47852/bonviewJDSIS42023640

[29] Alkraiji AI Jackson T Murray I. Factors impacting the adoption decision of health data standards in tertiary healthcare organisations in Saudi Arabia. J Enterp Inf Manag. (2016) 29:650–76. doi: 10.1108/JEIM-11-2014-0111

[30] Alkhwaldi AF. Understanding the acceptance of business intelligence from healthcare professionals’ perspective: an empirical study of healthcare organizations. Int J Organ Anal. (2024) 32:2135–63. doi: 10.1108/IJOA-10-2023-4063

[31] Kumar R Singh A Kassar ASA Humaida MI Joshi S Sharma M. Leveraging artificial intelligence to achieve sustainable public healthcare services in Saudi Arabia: a systematic literature review of critical success factors. Comput Model Eng Sci. (2025) 142:1289–349. doi: 10.32604/cmes.2025.059152

[32] Bell LC Shimron E. Sharing data is essential for the future of AI in medical imaging. Radiology. (2023) 6:337. doi: 10.1148/ryai.230337 PMC10831510 38231036

[33] Alasmari Y Alnowami MR Alkhateeb SM Djouider F. Assessment of radiographers’ understanding of radiation safety and their occupational radiation exposure in the Asir region of Saudi Arabia. Radiat Phys Chem. (2023) 212:111148. doi: 10.1016/j.radphyschem.2023.111148

[34] Choi HH Kotsenas AL Chen JV Bronsky C Roth CJ Kohli MD. Multi-institutional experience with patient image access through electronic health record patient portals. J Digit Imaging. (2022) 35:320–6. doi: 10.1007/s10278-021-00565-9, PMID: 35022926 PMC8921401

[35] Elzaki M Osailan R Almehmadi R Zulaibani A Kamal E Gareeballah A . Knowledge and comprehension of radiation protection among radiography professionals and interns in western Saudi Arabia. J Radiat Res Appl Sci. (2024) 18:101243. doi: 10.1016/j.jrras.2024.101243

[36] Gebbia V Piazza D Valerio MR Firenze A. WhatsApp messenger use in oncology: a narrative review on pros and cons of a flexible and practical, non-specific communication tool. Ecancermedicalscience. (2021) 15:1334. doi: 10.3332/ecancer.2021.1334, PMID: 35211203 PMC8816506

[37] Shen FX Wolf SM Lawrenz F Comeau DS Dzirasa K Evans BJ . Ethical, legal, and policy challenges in field-based neuroimaging research using emerging portable MRI technologies: guidance for investigators and for oversight. J Law Biosci. (2024) 11:lsae008. doi: 10.1093/jlb/lsae008, PMID: 38855036 PMC11157461

[38] Stratton SJ. Population sampling: probability and non-probability techniques. Prehosp Disaster Med. (2023) 38:147–8. doi: 10.1017/S1049023X23000304, PMID: 36939054

[39] Cha E Kim KH Erlen JA. Translation of scales in cross-cultural research: issues and techniques. J Adv Nurs. (2007) 58:386–95. doi: 10.1111/j.1365-2648.2007.04242.x, PMID: 17442038

[40] Taber KS. The use of Cronbach’s alpha when developing and reporting research instruments in science education. Res Sci Educ. (2018) 48:1273–96. doi: 10.1007/s11165-016-9602-2

